# Sedative and Anxiolytic Activities of *Cassia spectabilis* Leaf Extract: An *In Vivo* and *In Silico* Evaluation in a Mouse Model of Stress

**DOI:** 10.1155/vmi/7703472

**Published:** 2025-11-26

**Authors:** Wiwied Ekasari, Alfaniady I. Kurnia, Rahma K. Wirabuana, Vita M. A. Angembani, Elsa S. Prameswari, Windri A. A. Suri, Salsabilla Kristinawati, Tutik S. Wahyuni, Rico Ramadhan, Nindya T. Putri, Eko Suhartono, Ram K. Sahu

**Affiliations:** ^1^Department of Pharmaceutical Sciences, Faculty of Pharmacy, Universitas Airlangga, Surabaya, Indonesia; ^2^Department of Chemistry, Faculty of Science and Technology, Universitas Airlangga, Surabaya, Indonesia; ^3^Department of Medical Chemistry/Biochemistry, Faculty of Medicine and Health Science, Universitas Lambung Mangkurat, Banjarmasin, Indonesia; ^4^Department of Pharmaceutical Sciences, Hemvati Nandan Bahuguna Garhwal University (A Central University), Chauras Campus, Tehri Garhwal, India

**Keywords:** anxiolytic, *Cassia spectabilis*, depression, electric foot shock, sedative

## Abstract

Anxiety and depression are psychiatric disorders strongly associated with insomnia. This study aimed to examine the sedative and anxiolytic activities of a 70% ethanol leaf extract of *Cassia spectabilis* DC (CS70EE) in a mouse model with electric foot shock (EFS)-induced stress. Male BALB/c mice were subjected to mild EFS (1 mA) for 15 × 1 s for five consecutive days. The stressed mice were administered various doses of CS70EE, diazepam, and sodium carboxymethyl cellulose (0.5%) for comparison. The hole cross (HC) and thiopental sodium (TS)-induced sleep time method was used to assess sedative activity, while the elevated plus maze (EPM) test was used to assess anxiolytic activity. An in silico study was performed to predict the potential of active compounds in the extracts against sedative/anxiolytic target protein. The protein used is γ-aminobutyric acid receptor-associated protein (PDB code: 3D32). CS70EE at a dose of 500 mg/kg showed sedative activity in mice by significantly decreasing the number of passages through the hole in the HC test and increasing the TS-induced sleep duration (*p*  <  0.05). In the EPM test, stressed mice that were administered the extract (50 and 100 mg/kg) showed a significant increase in the time spent in the open arm (*p*  >  0.05). Meanwhile, molecular docking study in silico showed that spectaline compounds may play a crucial role in this activity. In conclusion, CS70EE exhibited sedative and anxiolytic effects in mice subjected to EFS-induced stress.

## 1. Introduction

Sleep is one of the physiological and psychological needs of humans, and almost one-third of a human life is spent sleeping. Therefore, sleep quality is one of the foundations of human mental and physical health. Sleep quality can be measured from the onset of sleep latency (the time taken to fall asleep), total duration of sleep, and number of awakenings from sleep at night. However, lifestyle changes and environmental factors cause sleep disorders, including insomnia [1]. It also causes anxiety and depression symptoms [2]. Insomnia can be managed with sedative-hypnotic drug therapy.

Sedative-hypnotic and anxiolytic drugs (often referred to as depressants) act by slowing brain activity through modulation of gamma-aminobutyric acid (GABA) receptors. Benzodiazepines and barbiturates are the main classes of these drugs. Although effective in managing anxiety, insomnia, and seizures, long-term use may lead to tolerance, dependence, and severe, which are sometimes fatal, withdrawal symptoms in barbiturate cases [3–6]. In addition, side effects such as memory and perception disturbances are also common [7, 8]. These limitations highlight the need for safer herbal alternatives for insomnia management.

*Cassia spectabilis* DC plant, known locally in Indonesia as *ramayana,* is found in many areas of Asia, Africa, Central America, and South America, where it is used as a shade plant. Phytochemical screening of various parts of *C. spectabilis* revealed the presence of different chemical groups such as piperidine alkaloids, pentacyclic triterpenes, steroids, pyrones, anthraquinones, and flavonoids. Two compounds have been isolated from the leaves of this plant, namely, (+)-spectaline and (−)-iso-6-cassine [9]. Phytochemicals isolated from other parts of the plant include spectalinine, iso-6-carnavaline [10], physcion, β-sitosterol, stigmasterol, 1,3,8-trihydroxy-2-methylanthraquinone, cassinicine [11], (−)-3-O-acetylspectaline, (−)-7-hydroxyspectaline, iso-6-spectaline, (−)-spectaline [12], (+)-3-O-feruloylcassine [13], α-amyrin, β-amyrin, ursolic acid, oleanolic acid, betulinic acid, lupeol, cycloeucalenol, friedelin, caffeine [14], luteolin, methoxyluteoline [15], chelidonic acid, and chrysophanol [16]. Previous studies have shown that this plant exhibits diverse pharmacological activities, including antibiofilm, antifungal, antibacterial, antioxidant, anti-inflammatory, antihyperalgesic, and antimalarial properties [17]. It also exhibits significant anticonvulsant activity and has the potential to inhibit seizures caused by epilepsy [18–20]. In the development of herbal medicinal products for the treatment of Alzheimer's disease, *C. spectabilis* and its piperidine alkaloid derivatives have shown significant cholinesterase inhibitory activity as well as moderate antioxidant activity [21–25].

Meanwhile, Bum et al. [18] and Nkantchoua et al. [19] reported that the working mechanism of *C. spectabilis* resembles that of hypnotic sedative drugs acting on GABA receptors. The thiopental sodium (TS)-induced sleep method is commonly used to screen compounds for hypnotic sedative effects, while the hole cross (HC) test assesses the effects of compounds on locomotor activity. Substances with sedative or central nervous system (CNS) depressant properties typically reduce the sleep onset time, prolong sleep duration, or both. Thus, combining these tests provides complementary information on potential sedative activity. In addition, the elevated plus maze (EPM) test is used to evaluate anxiolytic effects. Previous studies have shown that the 70% ethanol extract of *C. spectabilis* leaves (CS70EE) exerts CNS-depressant effects in normal mice [26]. However, its effects in mice exposed to electric foot shock (EFS)-induced stress remain unknown.

Therefore, this study aimed to evaluate the sedative and anxiolytic activities of CS70EE in mice subjected to EFS-induced stress using HC, TS-induced sleeping time, and EPM tests and to identify potential active compounds responsible for these effects through molecular docking analysis.

## 2. Materials and Methods

### 2.1. Plant Material and Preparation of Ethanolic Extract

Fresh *C. spectabilis* leaves were collected from the Purwodadi Botanical Gardens and identified by the Indonesian Institute of Sciences (LIPI), Pasuruan, Indonesia (No. 0371/IPH.06/HM/III/2019). As a reference, a herbarium voucher from *C. spectabilis* was maintained at the Pharmacognosy and Phytochemical Laboratory, Faculty of Pharmacy, University of Airlangga. Fresh leaves were washed and dried at a temperature of 25 ± 1°C. The leaves were pulverized into a fine powder, and 500 g of leaf powder was soaked in 70% ethanol (2.5 L) for 24 h. The leaf pulp was extracted twice, and the combined filtrates were concentrated using a rotary evaporator, yielding 55.7 g (11.14%) of crude 70% ethanol extract.

### 2.2. Animals

Male BALB/c mice (6–8 weeks, 25–35 g) were obtained from the Veterinary Pharma Center (PUSVETMA), Surabaya, Indonesia, and housed under standard environmental conditions (relative humidity 55%–65%; temperature 22 ± 2°C) and exposed to a 12-h light and dark cycle. They were acclimatized for 1 week prior to the experiment and had access to an appropriate supply of food and water ad libitum. All animal experimental procedures were approved by the Research Ethics Committee of the Faculty of Veterinary Medicine, Universitas Airlangga (Approval Nos. 2.KE.082.07.2021-2.KE.084.07.2021) and were conducted in accordance with institutional guidelines for the care and use of laboratory animals.

### 2.3. Chemicals and Drugs

The drugs used for the experiments in this report are diazepam (Kimia Farma Tbk., Jakarta, Indonesia), TS (PT. Bernofarm, Sidoarjo, Indonesia), and ketamine (KET-A-100, Agrovet, Peru). All drugs were freshly prepared and administered in 0.5% natrium carboxymethyl cellulose on each day of the experiment.

### 2.4. Stress Induction Procedure

An EFS-induced stress model was used in this study [27, 28]. The mice were placed in a Plexiglas chamber (24 × 29 × 40 cm) equipped with a grid floor made of stainless-steel rods. An electric shock from the shock generator was delivered with a light intensity of 1 mA for 1 sec. It was administered 15 times within an hour with a foot shock interval of 4 min. Stress induction was performed for five consecutive days before drug administration.

### 2.5. Drug Administration and Study Design

The experimental animals were randomly divided into six groups (*n* = 5 per group): a nonstress control group, stress control group, positive control group, and three treatment groups. Animals in the nonstress control group received 0.5% sodium carboxymethyl cellulose (Na CMC, 10 mL/kg) without being subjected to EFS stress, whereas those in the stress control group received 0.5% Na CMC (10 mL/kg) and had stress induced using EFS. Stressed animals in the positive control group were administered diazepam (1 mg/kg) for the HC- and TS-induced sleep time methods, whereas for the EPM test, they received diazepam (1.5 mg/kg). Mice in the three treatment groups were administered CS70EE at doses of 100, 250, and 500 mg/kg for the HC- and TS-induced sleeping time tests [18] and at doses of 10, 50, and 100 mg/kg for the EPM test [29]. Na CMC, diazepam, and plant extracts were applied orally once daily for 24 h after 5 days of stress induction. All animals were randomly assigned to treatment groups, and behavioral assessments were performed by an observer blinded to the group assignments to minimize bias.

### 2.6. Sedative Activity Test

#### 2.6.1. HC Test

The HC test was used to assess the sedative-like activity of CS70EE in mice subjected to EFS-induced stress [30, 31]. The instrument used was a wooden box without a roof, measuring 30 × 20 × 14 cm, partitioned in the middle with a fixed dividing wall, that has a hole (3 cm in diameter, 7.5 cm above the base). Immediately after drug administration, each mouse was placed on one side of the box. Movements through the hole from one compartment to the other were observed for 6 min. The number of passages through the hole was counted and recorded at 0, 30, 60, 90, and 120 min after the drug administration.

#### 2.6.2. TS-Induced Sleeping Time Test

The TS-induced sleeping time test was used to assess the sedative-like activity of CS70EE in mice with EFS-induced stress [32, 33]. Thirty minutes after drug administration, TS (60 mg/kg) was administered intraperitoneally to each mouse to induce sleep. Thereafter, the sleep onset latency (time between administration of TS and loss of the righting reflex) and sleep duration (time between loss and recovery of the righting reflex) in mice with EFS-induced stress were observed and recorded.

### 2.7. Anxiolytic Activity Test

#### 2.7.1. EPM Test

EPM was used to assess the anxiolytic-like activity of CS70EE in mice with EFS-induced stress [34]. The instrument used was a Labyrinth Plus, consisting of two open arms (30 × 6 cm) and two closed arms (30 × 6 × 15 cm), extending from the center area (6 × 6 cm) and elevated to 50 cm above the floor. Sixty minutes after drug administration, each mouse was placed in the center area facing one of the open arms, and its exploratory behavior was observed and recorded for 5 min. The number of arm entries and the time spent within each arm (open and closed) were recorded.

### 2.8. *In Silico* Molecular Docking

Ligands and target proteins were prepared using Chimera 1.15. We used (+)-spectaline (CID: 10019240), which are compounds presented in the leaves of *C. spectabilis*, and diazepam (CID: 3016) as ligands [9]. Meanwhile, GABA (GDP: 3D32) was used as the target protein [35, 36]. Diazepam in this study is a drug used as a comparison ligand to (+)-spectaline in order to determine the interaction of the two ligands with the target protein. Evaluation of absorption, distribution, metabolism, and excretion were analyzed using the Swiss ADME web server (http://www.swissadme.ch/index.php). For docking, we used tools from http://hdock.phys.hust.edu.cn/to generate docking and confidence scores. A more negative docking score indicates a greater binding potential between the ligand and the target protein. The confidence score represents the credibility level of binding between a ligand and target proteins. The docking results were visualized using the Biovia Discovery Studio 2024.

### 2.9. Statistical Analysis

Data are shown as the mean of three replicates and the standard error of the mean (SEM). The normality of data distribution was assessed using the Shapiro–Wilk test, and homogeneity of variance was evaluated using Levene's test to analyze the data. A one-way analysis of variance (ANOVA), followed by a post hoc test, was performed if the data were normally distributed and homogeneous. The Kruskal–Wallis or Mann–Whitney nonparametric test was used if the data were not normally distributed and homogeneous. The significant differences between groups were demonstrated through the *p* values that are lower than 0.05 (*p*  <  0.05).

## 3. Results

### 3.1. Sedative-like Activity of CS70EE in EFS-Induced Stressed Mice

#### 3.1.1. HC Test

The sedative-like activity of CS70EE was assessed using the HC test in EFS-induced stressed mice, and results are shown in Table 1. The number of passages through the hole decreased significantly after oral treatment of the plant extracts (100, 250, and 500 mg/kg) and diazepam (1 mg/kg). The greatest reduction in activity in the HC test occurred at the 30-min observation after the administration of plant extracts (500 mg/kg) and persisted until the fifth observation period (120 min) compared to the stress and nonstress control groups.

#### 3.1.2. TS-Induced Sleeping Time Test

Table 2 shows the sedative-like activity of CS70EE in EFS-induced stressed mice measured using the TS-induced sleep time method. Administration of diazepam (1 mg/kg) and plant extracts at doses 250 and 500 mg/kg significantly (*p*  <  0.05) reduced the time to sleep onset compared to the nonstress (289.25 ± 76.06 s) and stress (115.50 ± 2.90 s) control groups. In addition, a significant increase (*p*  <  0.05) in sleep duration was also observed in mice treated with diazepam (283.20 ± 52.11 min) and plant extracts at doses 250 and 500 mg/kg (249.00 ± 36.63 and 167.20 ± 13.38 min, respectively).

### 3.2. Anxiolytic-like Activity of CS70EE Against EFS-Induced Stress Mice

#### 3.2.1. EPM Test

**Figure 1:**
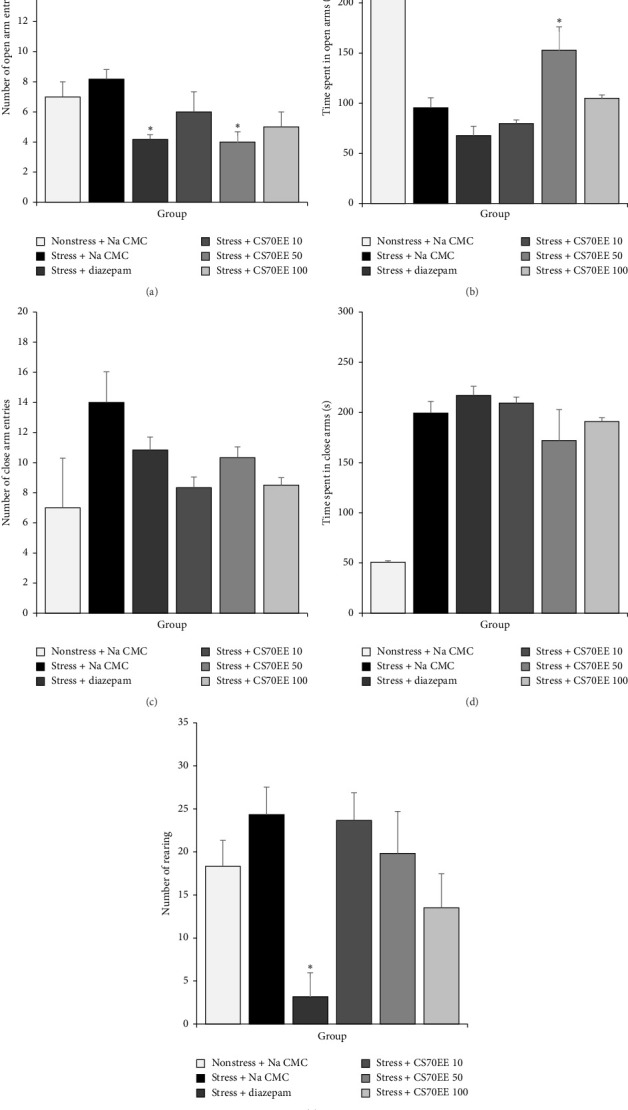
Effect of 70% ethanol extract of *C. spectabilis* leaves and diazepam on elevated plus maze test. (a) Number of open-arm entries during a 5 min exploration. (b) Time spent in open arms during a 5 min exploration. (c) Number of close-arm entries during a 5 min exploration. (d) Time spent in close arms during a 5 min exploration. (e) Number of rearing in both arms during a 5 min exploration. Data are expressed as mean ± SEM (*n* = 5 per group). ∗*p*  <  0.05 significantly different compared to stress control group. CS70EE = 70% ethanol extract of *C. spectabilis* leaves; the numbers refer to doses in mg/kg/day.

Administration of diazepam (1.5 mg/kg) and CS70EE (10, 50, and 100 mg/kg) did not change the number of open-arm entries in EFS-induced stressed mice compared to the stress and nonstress control groups (Figure 1(a)). However, the administration of plant extracts at the doses of 50 and 100 mg/kg was reported to increase the time spent in the open arm compared with the stress control group (Figure 1(b)). Additionally, treatment with CS70EE, particularly at 50 and 100 mg/kg, decreased the number of closed-arm entries (Figure 1(c)) and the time spent in closed arms (Figure 1(d)) while also reducing rearing activity (Figure 1(e)) compared with the stress control group, indicating attenuation of anxiety-like and stress-induced exploratory behaviors.

### 3.3. *In Silico* Molecular Docking Analysis for Sedative and Anxiolytic Properties

**Figure 2:**
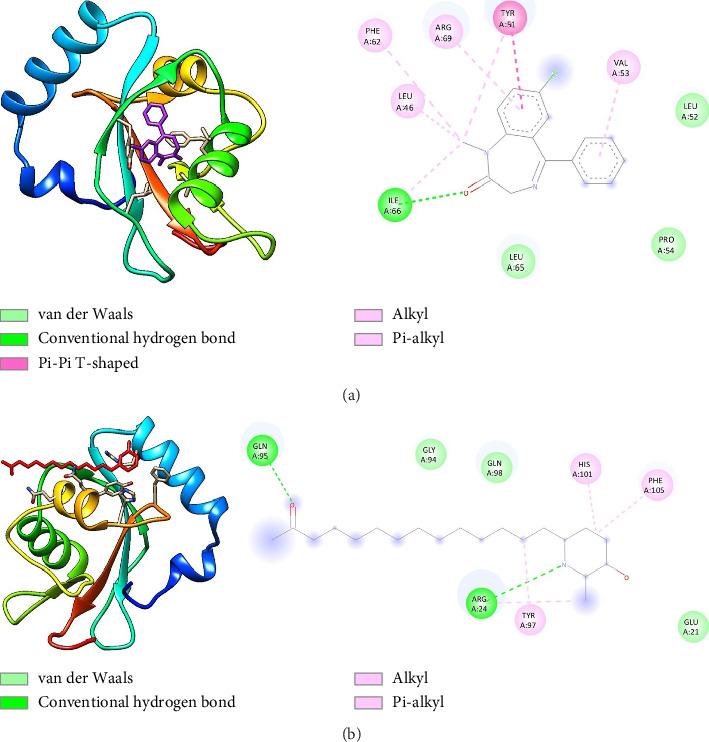
Interaction between GABA and (a) diazepam and (b) spectaline.

Molecular docking study on the effects of diazepam and spectaline on the target proteins is shown in Figure 2.

The docking and confidence scores of the diazepam and spectaline interactions with the target proteins are presented in Table 3.

As shown in Table 3, the docking score of GABA-spectaline was greater than that of GABA-diazepam, indicating that the binding potential of GABA-spectaline was better than that of GABA-diazepam. This is supported by the large number of hydrogen bonds in GABA-spectaline, namely, the ARG24 and GLN95 residues. The ADME results are presented in Table 4.

Based on Table 4, diazepam has high bioavailability and acts on the CNS but carries a high risk of drug–drug interactions due to inhibition of multiple CYP enzymes. Its toxicity potential is also greater in patients taking several medications simultaneously. In contrast, spectaline is well absorbed and able to cross the blood–brain barrier (BBB), with a safer metabolic profile since it does not strongly inhibit CYP enzymes. However, interactions with CYP2D6 substrates still need to be considered. Overall, spectaline is considered to have a safer potential compared to diazepam, particularly for the development of new drugs targeting the CNS.

## 4. Discussion

The sedative-like activity observed in mice treated with CS70EE, as indicated by reduced locomotor activity in the HC test and prolonged TS-induced sleep duration, suggests potentiation of inhibitory neurotransmission within the CNS. This effect is likely mediated through modulation of the GABAergic pathway, which plays a pivotal role in regulating neuronal excitability. Previous studies have demonstrated that *C*. *spectabilis* contains piperidine alkaloids such as (+)-spectaline and (−)-iso-6-cassine that interact with GABA_A_ receptor sites [18, 19, 37]. These findings align with earlier reports showing that extracts of *Senna spectabilis* produced anticonvulsant and CNS depressant effects through GABA facilitation in mice [18]. Such a mechanism parallels that of benzodiazepines, which enhance the frequency of chloride channel opening and induce sedation; however, plant-derived GABA modulators may offer a safer pharmacological profile with reduced risk of dependence. Comparable GABAergic-mediated sedative effects have also been reported for other botanical extracts, including *Opuntia ficus indica* [32] and *Glinus oppositifolius* [30], supporting the mechanistic consistency of our findings. The molecular docking results further support the experimental findings by demonstrating a stronger binding affinity of spectaline toward the GABA_A_ receptor compared with diazepam, as indicated by a more negative docking score and the formation of multiple hydrogen bonds. These computational results suggest that spectaline may act as a positive allosteric modulator of the GABA_A_ receptor complex, reinforcing its potential role in mediating the sedative and anxiolytic properties observed in vivo. This observation is in agreement with the previous pharmacological characterization of *C. spectabilis* and its piperidine alkaloid derivatives, which have shown inhibitory effects on seizure activity and enhancement of GABAergic neurotransmission [18, 19, 37]. The decision to focus the in silico analysis on spectaline was based on its predominance among the alkaloid constituents of *C. spectabilis* leaves [38] and its structural similarity to other GABA-active nitrogenous compounds. Nonetheless, considering that herbal extracts are chemically complex mixtures, it is plausible that the overall pharmacological effects arise from synergistic or complementary interactions among multiple constituents. Previous systems-pharmacology approaches have demonstrated that multicomponent herbal extracts can simultaneously target several neurotransmitter systems—including GABAergic, serotonergic, dopaminergic, and endocannabinoid pathways—yielding a broader neuropsychotropic profile [39, 40]. Such multitarget behavior may explain the overlapping sedative, antidepressant, and neuroprotective effects attributed to *C. spectabilis* in earlier studies [17, 24, 41]. Although docking analysis provides valuable molecular insights, it should be interpreted cautiously due to inherent limitations such as protein rigidity assumptions, simplified scoring algorithms, and the lack of dynamic physiological conditions. Therefore, complementary validation through in vitro receptor-binding assays and in vivo pharmacodynamic evaluations will be essential to confirm the biological relevance of these computational predictions.

To further clarify the pharmacological activity of CS70EE, we recommend expanding the behavioral test battery by including the rotarod test, a standard method for evaluating motor coordination and balance in rodents. As sedative agents may impair motor performance, incorporating this test would help differentiate between specific sedative or anxiolytic actions and nonspecific motor deficits. A decline in motor performance in this test would suggest that the reduced locomotor activity observed in the HC test may be partly attributable to motor impairment. A similar approach, combining multiple behavioral tests to evaluate sedative and anxiolytic effects, was employed by Shahed-Al-Mahmud and Lina [33], who assessed the methanolic extract of *Persicaria hydropiper* (MEPH) in mice. Their study showed that MEPH treatment significantly increased the number of falls and reduced motor performance time on the rotating rod. These effects were proposed to be mediated by mechanisms similar to those of certain benzodiazepines, such as diazepam, which induce muscle relaxation, reduce ambulatory movement, and cause sedation, thereby affecting motor performance on the rotarod.

In addition, the forced swim test (FST) should be considered as an additional assay, particularly in light of the EFS-induced stress paradigm used in this study. The FST is widely utilized to assess antidepressant-like effects and behavioral despair. The primary behavior evaluated in this test, termed “immobility,” reflects a depressed mood or helplessness experienced by animals when subjected to stressful situations, such as tail suspension or being forced to swim in a confined container from which escape is impossible. This behavior is considered indicative of a failure to maintain escape-directed behavior following persistent stress or the development of passive behavior that disengages the animal from active coping mechanisms [42]. As demonstrated by Martins and Brijesh [43], the bark extract of *Erythrina variegata* (Fabaceae) significantly reduced immobility duration when administered in both acute and chronic FST models. Effective antidepressants are known to reduce the duration of immobility observed in this behavioral screening model. Therefore, including the FST would allow for the evaluation of whether CS70EE exhibits broader psychoactive properties beyond sedation and anxiolysis, such as potential antidepressant effects. Incorporating both the rotarod and FST would provide a more comprehensive behavioral profile of CS70EE and strengthen the interpretation of its effects on the CNS.

A major source of bias is the limitation in protein flexibility; most docking methods assume that proteins are rigid, whereas under physiological conditions, proteins are dynamic. In addition, the scoring algorithms used to calculate binding affinity are often simplified and do not necessarily reflect complex biological environmental conditions, such as the presence of water, ions, or conformational fluctuations. This can lead to overestimation or underestimation of the potential biological activity of a ligand. In this study, Cavity-detection guided Blind Docking (CB-Dock) was used, which is one of the web servers used for automatic molecular docking with a blind docking approach—that is, docking without the need to determine the position of the binding site manually. CB-Dock is suitable as an initial screening tool before proceeding to more precise advanced docking or molecular dynamics simulation [44].

Stress induction using EFS with mild electrical power is likely to cause an increase in brain work in learning and memory, which is likely caused by the endogenous release of the neuropeptides angiotensin and angiotensin II, which are responsible for improving cognitive function during exposure to mild stress [27].

Mechanistic in vitro screening techniques, such as receptor binding assays, are highly selective and sensitive analytical methods [45, 46], making them suitable as experimental validation tools to confirm the docking potential of an active compound. In recent years, these techniques have become highly automated and transformed into high-throughput screening procedures, making them powerful tools in drug discovery research. Recent advances in synthetic organic chemistry—grouped under the general term “combinatorial chemistry”—have produced novel compounds that were previously unattainable and are now being evaluated through high-throughput screening procedures. Therefore, natural product research must take advantage of these emerging possibilities.

Additionally, receptor binding assays offer several clear advantages as first-line screening methods compared to in vivo pharmacometric screening [47, 48]. Scientifically, structure–activity data obtained from binding assays directly reflect ligand–receptor interactions with fewer complications from secondary events, bioavailability, or pharmacodynamic issues. Technically, binding studies require only a small quantity of test compound (≤ 1 mg), whereas whole-animal studies routinely require gram-scale quantities. Similarly, only a small amount of tissue is needed, in contrast to the higher costs and labor required for the purchase and maintenance of live animals in *in vivo* screenings. Supply costs and labor are greatly reduced due to the small volumes and test tube-based receptor binding technology. For these reasons, receptor binding assays have been widely and successfully used to identify lead compounds targeting specific sites across nearly all therapeutic areas, including, in the context of this study, to validate the potential of spectaline as a sedative and anxiolytic agent. However, due to time limitations in the current study, this receptor binding assay may be conducted in future research.

- The anxiolytic-like effect observed in the EPM test further supports the modulatory influence of CS70EE on central neurotransmission. The extract increased the time spent in open arms at moderate doses (50–100 mg/kg), suggesting a reduction in anxiety-related behavior without compromising motor performance. This dissociation between anxiolytic and sedative outcomes implies a dose-dependent pharmacological profile, where lower doses preferentially enhance anxiolysis while higher doses predominantly exert sedative effects. Such biphasic responses are characteristic of GABAergic modulators and have been reported for several plant-derived agents acting on similar pathways [30, 32]. The observed lack of effect on locomotor activity also indicates that CS70EE's anxiolytic action is not confounded by general sedation, thereby supporting its specificity for anxiety modulation. The mechanistic basis for this effect may involve partial agonism at the GABAA receptor or indirect facilitation of GABA release, mechanisms previously described for piperidine alkaloids of *C. spectabilis* [18, 19, 37]. Additionally, contributions from serotonergic and noradrenergic signaling pathways cannot be ruled out, as several alkaloid-containing extracts exert anxiolytic activity through simultaneous modulation of multiple neurotransmitter systems [17, 24, 41]. Interestingly, diazepam at the standard anxiolytic dose (1.5 mg/kg) did not significantly alter exploratory behavior in stressed mice, an observation that aligns with the findings of Pádua-Reis et al. [49], who reported strain- and dose-dependent variability in diazepam's effects. This apparent discrepancy underscores the complexity of interpreting behavioral data under stress paradigms such as EFS, which may alter baseline anxiety thresholds and receptor sensitivity. Taken together, these findings suggest that CS70EE exerts anxiolytic effects through a multifactorial mechanism that is partially independent of sedation and merits further mechanistic investigation under varying stress intensities.

Despite these promising findings, several limitations of the present study should be acknowledged. As the experiments utilized a crude ethanolic extract, variations in phytochemical composition may occur depending on plant source, harvesting season, and extraction parameters. Such variability could influence the pharmacological outcomes and reproducibility of the results. Moreover, the current investigation did not include acute or subchronic toxicity evaluations, which are essential for determining the safety margin and therapeutic window of CS70EE. Future studies should therefore incorporate comprehensive phytochemical standardization and toxicological profiling to establish a consistent quality framework for potential drug development. Another important direction for future research involves the isolation, purification, and structural modification of spectaline and its analogs to elucidate their structure–activity relationships and validate their pharmacodynamic mechanisms through receptor-binding and molecular dynamics assays. Integrating these approaches with advanced in vivo behavioral models—such as rotarod and FSTs—will further clarify the neuropsychopharmacological spectrum of *C. spectabilis*. Collectively, these efforts may advance the development of spectaline-based derivatives as safer and more selective GABAergic modulators for managing anxiety and sleep disorders.

## 5. Conclusion

The findings of this study demonstrate that the 70% ethanolic extract of *C. spectabilis* leaves (CS70EE) possesses significant sedative and anxiolytic activities in stress-induced mice models. These effects are likely mediated through GABAergic modulation, as supported by both behavioral and in silico evidence indicating a strong interaction of spectaline with the GABA_A_ receptor. The extract exhibited dose-dependent effects, producing anxiolysis at moderate doses and sedation at higher doses, suggesting a dual pharmacological profile characteristic of GABAergic agents.

Overall, *C. spectabilis* represents a promising natural source of GABA-modulating compounds with potential therapeutic applications for anxiety and sleep disorders. However, further research is warranted to isolate and characterize its active alkaloid constituents, particularly spectaline derivatives, and to establish their safety, efficacy, and precise mechanisms of action through standardized preclinical and toxicological studies. Future work may also incorporate behavioral assays such as the rotarod and FSTs to further evaluate motor coordination and antidepressant-like effects, thereby providing a more comprehensive pharmacological assessment of CS70EE.

## Figures and Tables

**Table 1:** Effect of 70% ethanol extract of *C. spectabilis* leaves (CS70EE) on hole cross test.

Treatment (mg/kg)	Number of passages through the hole				
	0 min	30 min	60 min	90 min	120 min
Nonstress control	3.60 ± 3.56	4.00 ± 1.10	6.40 ± 3.44	4.00 ± 2.61	1.00 ± 0.45
Stress control	0.40 ± 0.40	3.00 ± 1.55^a^	2.40 ± 0.25	4.00 ± 2.26	1.20 ± 0.97
Diazepam 1	0.20 ± 0.20	2.80 ± 1.20^a^	0.00 ± 0.00	1.00 ± 0.55	0.40 ± 0.40
CS70EE 100	2.60 ± 2.60	4.00 ± 2.10^a^	3.40 ± 1.43^a^	3.00 ± 1.48^a^	1.80 ± 1.56
CS70EE 250	0.00 ± 0.00	2.80 ± 0.80	4.00 ± 1.71^a^	2.20 ± 1.71	1.40 ± 0.60^a^
CS70EE 500	1.00 ± 1.00	1.20 ± 0.80	0.40 ± 0.40	0.80 ± 0.81	0.00 ± 0.00

*Note*: Data are expressed as mean ± SEM (*n* = 5 per group). Statistical analysis was performed by one-way ANOVA followed by post hoc test. CS70EE = 70% ethanol extract of *C. spectabilis* leaves.

^a^ *p*  <  0.05 indicates significant difference compared with the nonstress control group.

**Table 2:** Effect of 70% ethanol extract of *C. spectabilis* leaves (CS70EE) on thiopental sodium-induced sleep time test.

Treatment (mg/kg)	Sleep onset latency (s)	Duration of sleep (min)
Nonstress control	289.25 ± 76.06	99.20 ± 26.01
Stress control	115.50 ± 2.90	81.80 ± 20.10
Diazepam 1	88.25 ± 5.57^a^	283.20 ± 52.11^a^
CS70EE 100	118.50 ± 6.25	148.80 ± 17.20^a^
CS70EE 250	93.25 ± 5.39^a^	249.00 ± 36.63^a^
CS70EE 500	96.00 ± 5.35^a^	167.20 ± 13.38^a^

*Note*: Data are expressed as mean ± SEM (*n* = 5 per group). Statistical analysis was performed by one-way ANOVA followed by post hoc test. CS70EE = 70% ethanol extract of *C. spectabilis* leaves.

^a^ *p*  <  0.05 indicates significant difference compared with the nonstress control group.

**Table 3:** Binding site analyses of best ligand and receptor interactions by the HDOCK server.

	GABA-diazepam	GABA-spectaline
Docking score	−96.53	−99.08
Confidence score	−0.2555	0.2653
Interaction		
Hydrogen bonding	ILE66	ARG24; GLN95
Hydrophobic bonding	LEU52; PRO54; LEU65	GLU21; GLY94; GLN98
π-π	TYR51	—
Alkyl	LEU46; PHE62; ARG69; VAL53	TYR97; HIS101; PHE105

**Table 4:** ADME analysis of diazepam and spectaline.

Properties	Diazepam	Spectaline
GI absorption	High	High
Permeant BBB	Yes	Yes
P-gp substrate	No	No
CYP1A2 inhibitors	Yes	No
CYP2C19 inhibitors	Yes	No
CYP2C9 inhibitors	Yes	No
CYP2D6 inhibitors	Yes	Yes
CYP3A4 inhibitors	Yes	No
Log Kp (skin permeation)	−5.91 cm/s	−4.58 cm/s

## Data Availability

Data used to support the findings of this study are available from the corresponding author upon request.
